# Dexmedetomidine is effective and safe during NIV in infants and young children with acute respiratory failure

**DOI:** 10.1186/s12887-018-1256-y

**Published:** 2018-08-25

**Authors:** M. Piastra, A. Pizza, S. Gaddi, E. Luca, O. Genovese, E. Picconi, D. De Luca, G. Conti

**Affiliations:** 1 Pediatric Intensive Care Unit, Fondazione Policlinico A. Gemelli IRCCS and Catholic University of Rome, L.go A.Gemelli, 8, Rome, Italy; 2 Division of Pediatrics and Neonatal Critical Care, Medical Center “A.Béclère”, South Paris University Hospitals, Paris, France

**Keywords:** Sedation, Non invasive ventilation, Infant, Dexmedetomidine, Acute respiratory failure

## Abstract

**Background:**

Noninvasive ventilation (NIV) is increasingly utilized in infants and young children, though associated with high failure rates due to agitation and poor compliance, mostly if patient-ventilator synchronization is required.

**Methods:**

A retrospective cohort study was carried out in an academic pediatric intensive care unit (PICU). Dexmedetomidine (DEX) was infused as unique sedative in 40 consecutive pediatric patients (median age 16 months) previously showing intolerance and agitation during NIV application.

**Results:**

During NIV clinical application both COMFORT-B Score and Richmond Agitation-Sedation Scale (RASS) were serially evaluated. Four patients experiencing NIV failure, all due to pulmonary condition worsening, required intubation and invasive ventilation. 36 patients were successfully weaned from NIV under DEX sedation and discharged from PICU. All patients survived until home discharge.

**Conclusion:**

Our data suggest that DEX may represent an effective sedative agent in infants and children showing agitation during NIV. Early use of DEX in infants/children receiving NIV for acute respiratory failure (ARF) should be considered safe and capable of improving NIV, thus permitting both lung recruitment and patient-ventilator synchronization.

## Background

Noninvasive ventilation (NIV) has been reported as effective for treatment of respiratory failure associated with different respiratory diseases in childhood [1, 2]. Its usefulness has also been suggested in postoperative and immunocompromised pediatric patients [3, 4]. However, interface intolerance and agitation may represent a major clinical problem and a frequent cause of NIV failure. Some authors have reported the use of sedatives to obtain adequate compliance with NIV. Although NIV requires less sedation than invasive ventilation, agitated patients should be given the minimum sedation necessary to tolerate NIV interfaces.

Despite in adults data suggesting that the use of sedatives or opioids may improve patient comfort and tolerance during NIV, [5–7]. Pediatric intensive care unit (PICU) clinicians are often reluctant to administer these drugs in non-invasively ventilated infants, mainly because of concerns that they may induce respiratory and cardiovascular side effects [8–10].

Dexmedetomidine (DEX) is an α2-adrenergic agonist with a unique mechanism of action, providing sedation and anxiolysis via receptors within the locus coeruleus, analgesia via receptors in the spinal cord, and attenuation of stress response with no significant respiratory depression; several studies have demonstrated short length of weaning [8–11]; Moreover, DEX has been recently proposed to manage NIV failure due to interface intolerance in adult patient with acute respiratory failure (ARF).

To date, few data have been reported on the use of DEX as a single agent for sedation in pediatric patients showing agitation during NIV. In this retrospective study, we describe our experience with DEX as a single sedative agent during NIV in pediatric patients.

## Methods

### Setting

This retrospective uncontrolled clinical study was conducted at Catholic University PICU. The Institutional Review Board approved the study and waived the need for a written informed consent as DEX was part of the standard sedative regimen in our PICU. According to our national legislation, intravenous DEX is allowed in pediatric patients undergoing ventilatory support within a ICU setting.

### Patients

From january 2013 to july 2014, 40 consecutive infants and children admitted to our PICU with ARF and managed with NIV for > 8 h were evaluated. Indication for NIV was: early onset dyspnea, typical findings on chest radiograph (e.g., marked hyperinflation, bilateral infiltrates, perihilar bat wing appearance), and signs of acute respiratory distress defined by mild-to-moderate hypoxemia (Pao2/Fio2 > 100 < 300 mmHg). Patients were deemed as needing sedation during NIV if they were uncooperative due to young age, with 1 or more on the RASS score and 22 or more on the Comfort-B scale [12, 13].

Exclusion criteria were: age > 12 years, systolic blood pressure (BP) < 80 mmHg, heart rate < 60 beats/min, the presence of acute decompensated heart failure accompanied by a left ventricular ejection fraction < 25%, heart block of every grade, hepatic or renal failure, digestive tract hemorrhage or a do-not-resuscitate or do-not-intubate order.

NIV was performed using a Maquet-Servo I ventilator with NIV software, whereas CPAP was administered by a Drager Continuous flow CPAP generator. NIV failure was defined, according to our PICU protocol, as the need for intubation and invasive ventilation.

The primary endpoint of the study was the efficacy of sedation with DEX during NIV, as demonstrated by the sedation scores.

Secondary endpoints were a) the improvement of gas exchanges while on NIV, b) the rate of NIV failure due to NIV intolerance, b) the rate of DEX-related cardiovascular side effects.

### Infusion protocol

All patients started DEX at a 0.5–0.7 mcg/kg/h intravenous continuous infusion and titrated upwards until 1.0–1.4 mcg/kg/hr. depending on the sedation level achieved. All patients during NIV were maintained within an adequate sedation target assessed by the Comfort-B score between 11 and 22. DEX maximal dose was 1,4 mcg/kg/h. In all cases, no bolus of DEX was administered.

### Sedation state assessment and data collection

The level of sedation was evaluated as the main outcome variable using the Comfort-B score and RASS. These scores were evaluated at baseline and after 2, 8, 16,24,48,72 h from starting sedation, according to our PICU NIV protocol. Comfort-B scale [12, 13] is designed for infants and children, containing 6 assessment categories: level of consciousness; agitation; respiratory response (if patient is under mechanical ventilation) or crying; physical movements; muscular tone and facial tension. Each category can take a score from 1 to 5 for a global score from 6 to 30. According to our institutional protocol a score under 10 describes an over-sedated is over-sedated, above 23 describes an under-sedated patient. Between 11 and 16 the sedation is optimal, while between 17 and 23 the sedation is uncertain, the patient could feel pain. Comfort-B and RASS scores [14, 15] were evaluated by the attending nurse on a regular basis and inserted in the nurse section of the PICU electronic chart.

All the cardio-respiratory variables were retrospectively collected from our electronic medical records software. (Digistat®, Florence, Italy).

Data were collected using a Microsoft Excel 97–2003 spreadsheet (Microsoft Corporation, Redmond, USA) and analyzed in SPSS version 20.0 (IBM, Armonk, *USA*). Mean, median, SD and Inter Quartile Range (IQR) are given for normally distributed metric variables, frequencies and percentages are given for non-metric variables. T-Test or Mann-Whitney U tests were performed, as appropriate; Fisher’s exact test or Pearson’s chi square were applied to observe associations for qualitative variables. A *p*-value of < 0.05 was considered statistically as significant.

## Results

Median age was 16 months (IQR 6,5; 33.50), while median body weight was 12 kg (IQR 6,2; 17) and M/F ratio 3.0; main diagnoses were bronchiolitis [12]; Acute respiratory distress syndrome (ARDS) [7] chest trauma [2], burn-associated respiratory failure [4], status asthmaticus [3], neurological illness [3], pneumonia [4], Bronchopulmonary Dysplasia [3] and post-operative patients [2]. Median Pediatric Risk of Mortality (PRISM)-III24 for the study group was 15.5 (IQR 12;22) (Table 1).

**Table 1:** Demographic data (*n* = 40 patients)

Variables	Median (IQR)
Age (months)	16 (6,50-33,50)
Weight (kg)	12 (6,2–17)
Gender (M/F)	30/10
PRSIM III _24_	15,50 (12–22)
Niv Duration (hours)	48 (36;96)
PICU LOS (days)	7 (5–18,7)
PO_2_/FiO_2_ Ratio at NIV onset	175 (150–203)
Main diagnosis	
Bronchiolitis	12 (30%)
ARDS	7 (17,5%)
Chest trauma	2 (5%)
Burn-associated Respiratory Failure	4 (10%)
Status Asthmaticus	3 (7,5%)
Neurological illness	3 (7,5%)
Pneumonia	4 (10%)
Bronchopulmonary Dysplasia	3 (7,5%)
Post-operative patients	2 (5%)
NIV Interfaces	
Helmet	12 (30%)
Total Face Mask	11 (27,5%)
Nasal Mask	17 (42,5%)

*NIV* Non Invasive Ventilation, *PICU LOS* Pediatric intensive Critical Unit length of stay, *ARDS* acute respiratory distress syndrome

**Fig. 1:**
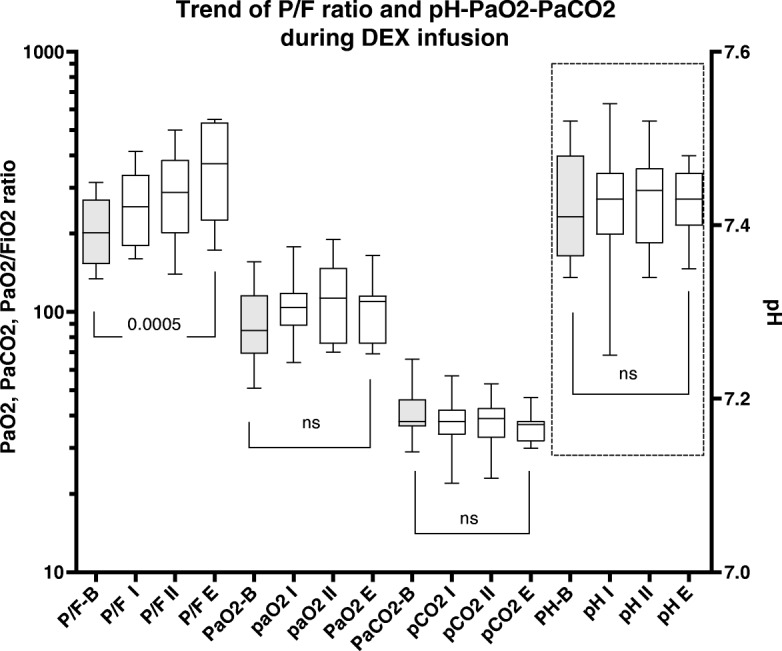
Arterial gas analysis and respiratory parameters during NIV in DEX. P/F: PaO2 /FiO2 ratio; B: Basal; I: after 8 h; II; after 24 h; E (End): after 48 h

**Fig. 2:**
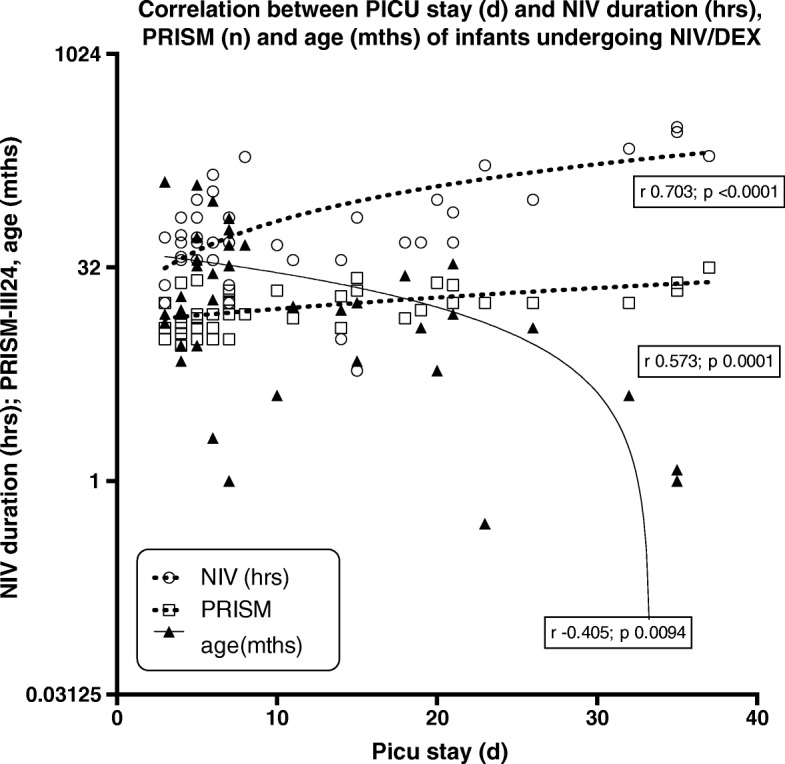
Correlation between PICU stay (d) and NIV duration (hrs), PRISM (n) and age (in months) of infants undergoing NIV during DEX sedation

All patients were hypoxemic at NIV beginning, with a median P/F ratio of 175 (IQR 150;203). NIV was associated with a significant P/F ratio increase (Fig. 1). Median NIV application was 48 h (IQR 36; 96), with a median PICU stay of 7 days (IQR 5.0; 18.7); as a whole, 16 (40%) patients received Continuous Positive Airway Pressure (CPAP) only, while the remaining patients were given Non Invasive Positive Pressure Ventilation (NPPV) (12 patients, 30%) and NPPV+CPAP (12 patients, 30%). NIV was administerd in 12 patients using a helmet (30%), a TotalFaceMask in 11 pts. (27,5%) and a Nasal Mask in 17 pts. (42,5%). PICU lenght of stay (LOS) was statistically related to the duration of NIV and to the severity score (PRISM-III24) and inversely related to patients’s age (Fig. 2).

**Fig. 3:**
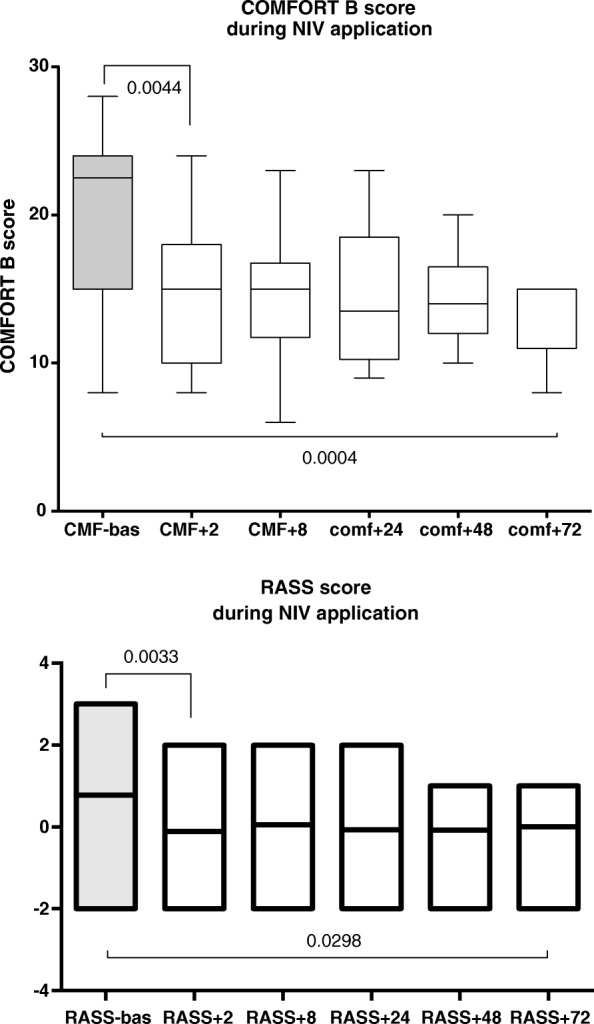
Trend in HR (bpm) and MAP (torr) in infants undergoing NIV

With regard to the *primary study endpoint*, Comfort-B score and RASS significantly decreased from the basal value, assessed just before starting sedation (Fig. 3). A significant difference between Comfort-B scale at the basal value and 2 h after DEX infusion (*p*: 0,001) was recorded; then the sedation’s degree remained stable.

No patient required NIV discontinuation due to NIV intolerance: 4 infants (10%) required NIV suspension and conversion to endotracheal intubation and conventional ventilation, due to a progressive deterioration of their respiratory condition; all these patients were shifted back to NIV and could be weaned and discharged from the PICU.

**Fig. 4:**
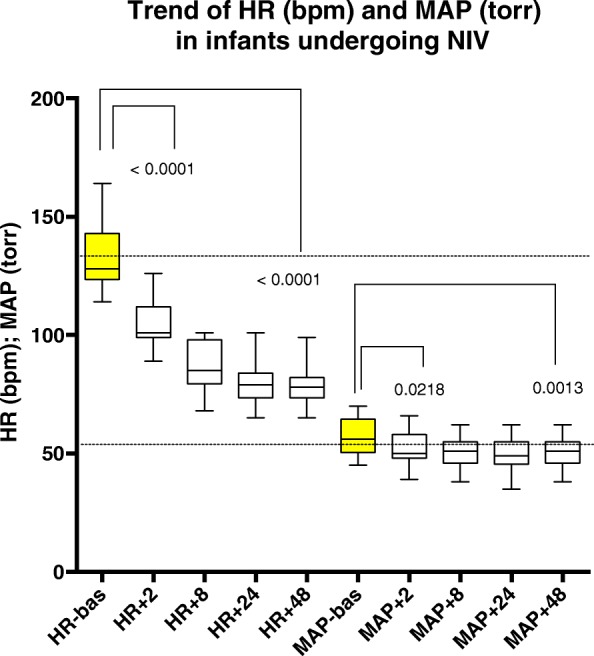
COMFORT B score and RASS score during NIV application

DEX infusion was associated with cardiocirculatory modifications, as evidenced in Fig. 4. A significant decrease in heart rate (HR) and mean arterial pressure (MAP) after 2 h from DEX introduction was recorded. Conversely no significant differences in heart rate (HR) and MAP were observed in subsequent time-points during the infusion of DEX. Regarding the effect on heart rate, pre-NIV application median levels for HR were 128 bpm (IQR 123.5; 143). No patient developed severe bradycardia or hypotension requiring DEX infusion interruption or rescue drugs.

## Discussion

The results of this retrospective analysis suggest that the use of DEX is effective, safe and associated with NIV tolerance, with a low level of NIV failure in infants and young children.

There is increasing interest in NIV use in critically ill infants and children, as NIV can reduce the intubation rate [1] improving gas exchange [16] and decreasing work of breathing (WOB) [17].

On the other hand, NIV failure has been associated with a high risk of prolonged mechanical ventilation [18]. Published rates of NIV failure (i.e., patients intubated after starting NIV) range between 8.8 and 43%, depending on the population under assessment [19, 20].

Patient cooperation is crucial for NIV success at all ages, particularly if patient-ventilator synchronization is required, as during NPPV. As a consequence, young children often show a suboptimal interaction, having both higher breathing rates [21] and less effective inspiratory efforts than adults, with increased asynchronies. Recent data have shown the association of an high rate of asynchrony with increased NIV failure rate and ventilatory support prolongation [22, 23].

Mask intolerance generated by pain and discomfort may lead the refusal of NIV, prompting its discontinuation and leading to endotracheal intubation [24]; Conversely, gas exchange can improve under NIV when an adequate level of anxiolysis and analgesia are obtained, resulting in better synchronization and patient-ventilator interaction; Patient-Ventilator synchronization per se further improves patient tolerance [25].

Due to poor cooperation and anticipatory anxiety, NIV before 2 years of age is often achievable only as CPAP or unsynchronized NPPV [21]. This condition is likely to limit the field of application of NIV to the less severely ill patients.

In the adult experience, few studies suggest that continuous infusion of a single sedative agent – as a benzodiazepine or an opioid - may decrease patient discomfort, with no significant effects on respiratory drive, respiratory pattern, or hemodynamics [26, 5, 7, 27].

Few data exist regarding current sedation practices during NIV in childhood, and no specific investigation has been reported. All recent publications about sedation in pediatric NIV refer to common practices rather than controlled studies [28–30].

Among the sedatives, DEX may offer an optimal sedation profile, for its low risk of depression of the respiratory centres, associated with unmodified airway patency [31].

In PICU, prolonged DEX infusions have been associated with a reduction in concomitant analgesics and sedatives. A recent study demonstrated that DEX used as primary sedation agent or in peri-extubation time improves the quality of sedation compared to other sedatives [32]. DEX has been reported diffusely as well tolerated; also cardiovascular variables remained within clinical acceptable ranges, provided bolus doses were avoided [33]. DEX commonly reported side effects are hypotension and bradycardia, but have been inconsistently reported among critically ill children. Tobias and Berkenbosch [34], in their randomized controlled trial, did not report clinically significant hypotension but one patient did develop bradycardia requiring DEX discontinuation. Walker et al. [35] did not report any adverse effects. Both Chrysostomou et al. [36] and Diaz et al. [37] found that hypotension occurred more frequently than bradycardia, requiring either a decrease or complete cessation of DEX infusion.

### Limitations of the study

Some limitations of this study must be acknowledged: the most important is its retrospective design, suggesting the need for confirmatory data from prospective controlled studies. Secondly, this study was performed in a PICU with large experience both on NIV and DEX use, thus possibly reducing the generalization of the obtained results.

## Conclusions

The results of our study suggest that DEX infusion may provide an effective light level of sedation (i.e Comfort-B between 10 and 22 and RASS above 1) in infants and young children receiving NIV, avoiding the association with opioids or benzodiazepines.

In infants receiving NIV, the possibility of maintaining a regular respiratory activity without depression episodes and/or upper airway obstruction is of great relevance. In our experience, the use of DEX sedation was safe and well tolerated, permitting the successful application of NIV in this young pediatric population.

## Abbreviations

ARF: Acute respiratory failure
BP: Blood pressure
CPAP: Continuous Positive Airway Pressure
DEX: Dexmedetomidine
HR: Heart rate
IQR: Inter Quartile Range
LOS: PICU long of stay
MAP: Mean arterial pressure
NIV: Noninvasive ventilation
NPPV: Non Invasive Positive Pressure Ventilation
PICU: Pediatric intensive care unit
PRISM: Median Pediatric Risk of Mortality
RASS: Richmond Agitation-Sedation Scale
